# Coexistence of two talon cusps and two dens invaginatus in a single tooth with associated radicular cyst-a case report and review of literature

**DOI:** 10.4317/jced.51421

**Published:** 2014-10-01

**Authors:** Vikrant O. Kasat, Mukund Singh, Harish Saluja, Ruchi Ladda

**Affiliations:** 1 MDS, Associate Professor. Department of Oral Medicine and Radiology, Rural Dental College, Loni; 2 MDS, Assistant Professor. Department of Conservative Dentistry, Rural Dental College, Loni; 3 MDS, Assistant Professor. Department of Oral and Maxillofacial Surgery, Rural Dental College, Loni; 4 MDS, Assistant Professor. Department of Prosthodontics, Rural Dental College, Loni

## Abstract

Talon cusp (TC) and dens invaginatus (DI) are relatively rare developmental anomalies which affect the shape of teeth. TC is an additional cusp that projects predominately from the lingual surface of anterior teeth. DI is a deep surface invagination of the crown or root which is commonly detected on routine radiographic examination. Both of these anomalies are observed more frequently in permanent maxillary lateral incisor. Isolated cases of single TC and DI are extensively reported in dental literature. Occasionally two talon cusps (TCs) in the same tooth as well as double and triple invaginations in one tooth are reported separately. Coexistence of these two anomalies in a single tooth is considered extremely rare, but still there are few reported cases. However, coexistence of two TCs and double DI in a single tooth is not reported till date. We hereby report the first case of coexistence of two TCs and double DI in a single tooth of a 23 year old female and use of platelet-rich fibrin (PRF) in the management of associated radicular cyst. Also, literature on coexistence of these two anomalies in a single tooth is reviewed.

** Key words:**Coexistence, dens invaginatus, radicular cyst, talon cusp.

## Introduction

TC is a rare developmental anomaly of teeth which was first recognized in 1892 by Mitchell (1). Later on, Mellor and Ripa in 1979 gave the name talon because its shape appeared similar to that of an eagle’s talon (1). It is an additional cusp that projects predominately from the lingual or occasionally from the labial surface of anterior teeth (2,3). It is more common in males than females (2) and reported prevalence ranges from less than 1% to approximately 8% of the population (1,4). It mainly affects the permanent dentition (1,4) and majority of the cases [92%] are seen in the maxillary teeth (3) where it predominantly occurs in lateral incisors [55%] followed by central incisors [32%] and canines [9%] (1,3,4).

DI is another rare developmental anomaly of teeth which was first described by a dentist named ‘Socrates’ in 1856 (5). It is a deep surface invagination of the crown or root which is lined by enamel (2). The reported prevalence varies from 0.04% to 10% of the population (1,2,5). It is seen predominantly in the maxilla and the most commonly affected tooth is the permanent maxillary lateral incisor (5), followed by the central incisor (1), premolar, canine and molar (2). DI is commonly detected on routine radiographic examination as an infolding of enamel and dentine extending into the pulp cavity or the root or sometimes even reaching the root apex (5).

Isolated cases of single TC and DI have been reported extensively in the dental literature. Rarely, two or more TCs occur in a single tooth. Sarraf-Shirazi (1) reported two palatal and one labial TCs in maxillary right central incisor. Similarly double and triple DI in one tooth is also reported occasionally in the literature (5). Though coexistence of TC and DI within the same tooth is considered a rarity (6), 8 such cases have been reported so far in the English literature as revealed by PUBMED search ( Table 1). However, coexistence of two TCs and double DI in a single tooth is not reported till date. Therefore, the purpose of this paper is to report the first case of coexistence of two TCs and double DI in a single tooth of a 23 year old female patient and discuss management of associated radicular cyst.

**Table 1:**
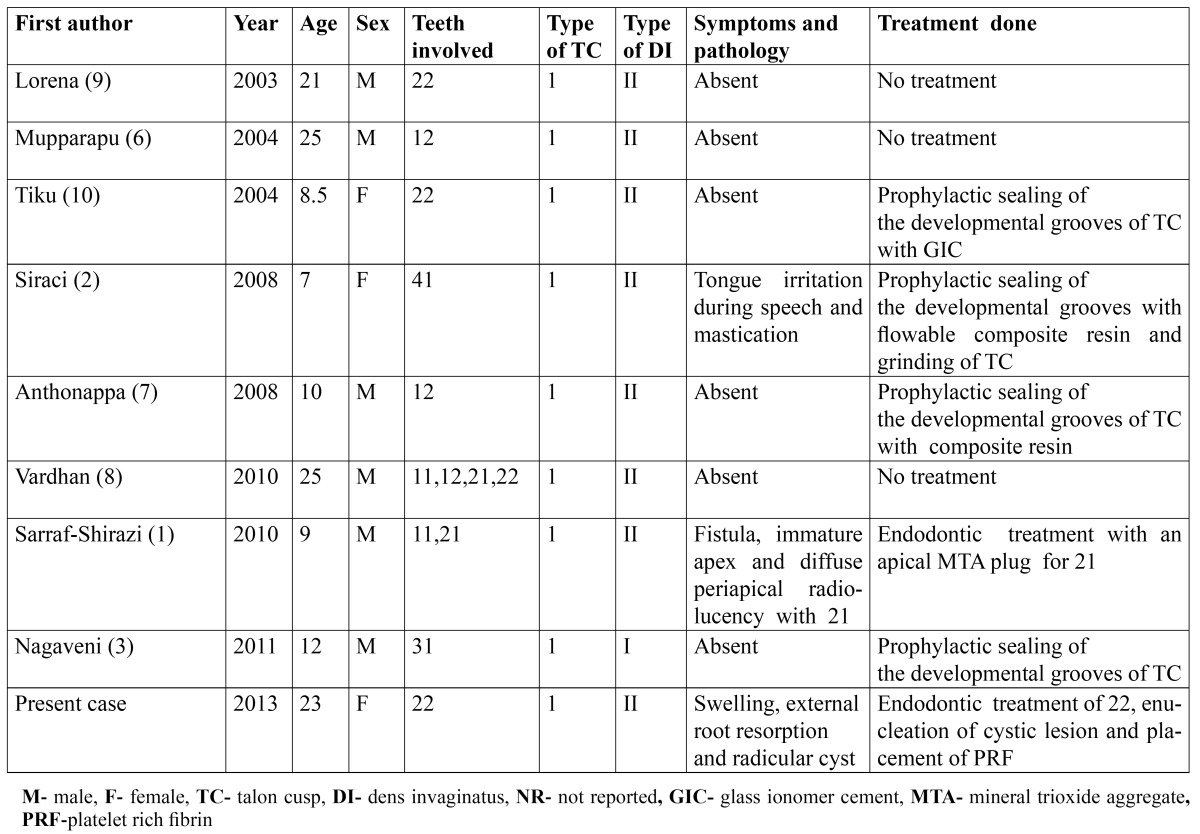
Summary of case reports of concurrent occurrence TC and DI in chronological order.

## Case Report

**Figure 1:**
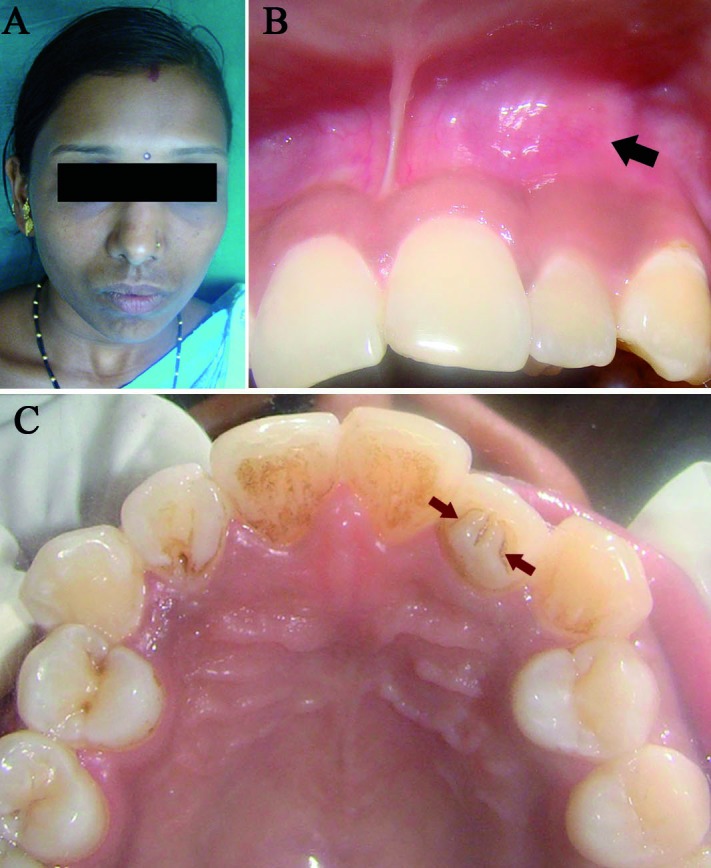
A) Extra- oral photograph; B) Intra-oral view showing diffuse swelling in labial vestibule (black arrow); C) Palatal view showing two talon cusps (brown arrows).

A 23 year old female reported with the complaint of recurrent swelling in the upper left anterior region of jaw since last 4-5 months. Her medical and family history was noncontributory and general physical examination revealed no other abnormalities. On intraoral examination a diffuse, firm and tender swelling was seen in the labial vestibule in relation to maxillary left lateral incisor. Loss of labial vestibular depth was noted. The overlying mucosa was pink in colour and had smooth surface (Fig. 1). Involved tooth didn’t show signs of mobility or tenderness. On palatal surface of lateral incisor, two triangular projections were seen which were separated by a notch. They were extending from the cingulum towards the incisal edge and covering more than half of the crown length (Fig. 1). A provisional diagnosis of two TCs in lateral incisor with associated acute exacerbation of chronic periapical infection was made.

**Figure 2:**
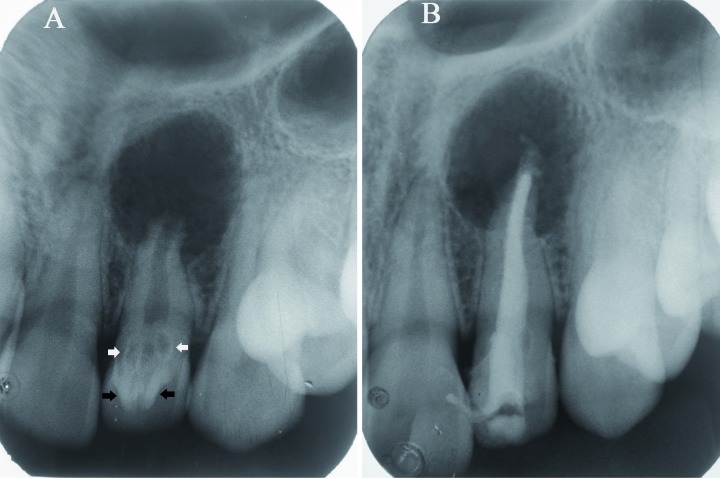
A) Intraoral periapical radiograph (IOPA) showing two dense invaginatus (white arrows) below two talon cusps (black arrows) and a well defined radiolucency in relation to 22; B) Post-obturation radiograph.

Intraoral periapical radiograph [IOPA] of the lateral incisor was taken (Fig. 2) which revealed two V-shaped radiopaque TCs in the crown [black arrows]. Apical to it, two key-hole shaped radiolucencies with radiopaque border were seen extending below CEJ in the coronal 1/3rd of root [white arrows]. Loss of lamina dura, external root resorption and open apex with lateral incisor were noted. A well defined, partly corticated radiolucency was seen in the periapical area which was approximately 1x1 cm in diameter. Correlating clinical and radiographic findings, a diagnosis of coexistence of two TCs and double DI in lateral incisor with associated radicular cyst was made.

**Figure 3:**
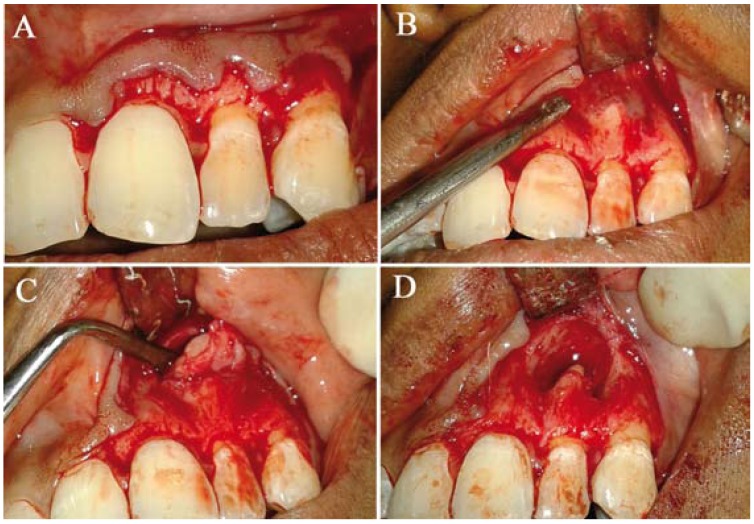
A-D) Intra-operative view of enucleation of lesion.

**Figure 4:**
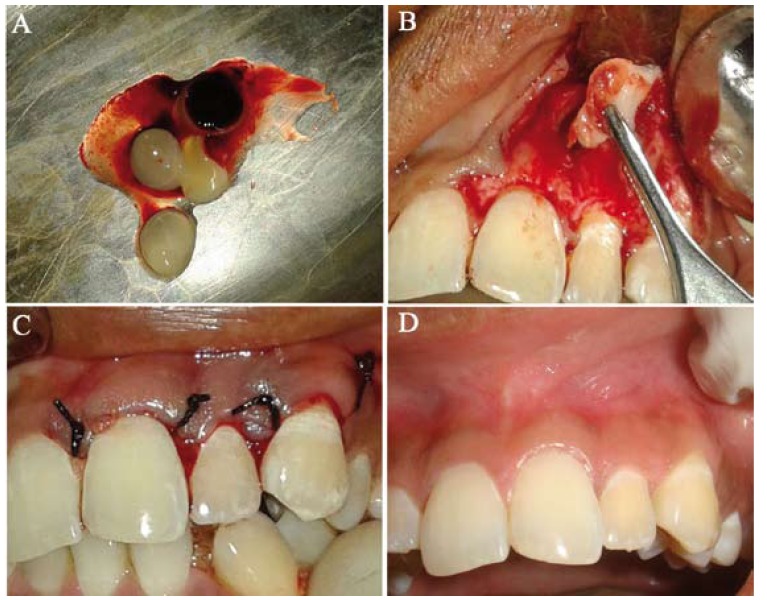
A-B) Placement of platelet rich fibrin in the bony cavity; C) Post operative photograph; D) 4 week follow up photograph.

**Figure 5:**
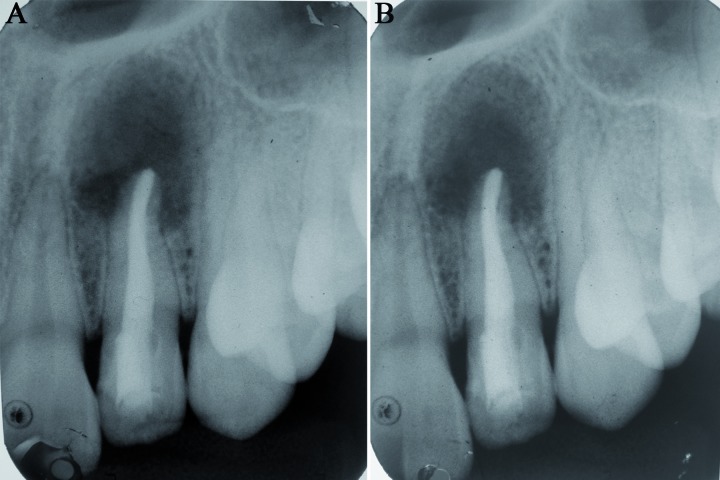
A) 6 week follow up radiograph; B) 12 week follow up radiograph showing bone formation from periphery to center.

After performing root canal treatment on the lateral incisor (Fig. 2), enucleation of the cystic lesion was carried out under local anesthesia (Fig. 3) and bony cavity was filled with platelet rich fibrin (Fig. 4). Enucleated tissue was sent for histopathological examination which confirmed the diagnosis of radicular cyst. Postoperative healing was uneventful (Fig. 4). Patient was followed up after 4, 6, 12 weeks and radiographs revealed healing surgical defect (Fig. 5). Also, all ceramic crown was given on the lateral incisor.

## Discussion

The exact etiology of TC and DI is still not well understood. Simultaneous occurrence of these two anomalies in a single tooth suggests common etiological factor. TC originates during the morpho-differentiation stage of tooth development and may occur as a result of abnormal proliferation of inner enamel epithelial cells and transient focal hyperplasia of the peripheral cells of mesenchymal dental papilla (2-4,6-8). Both genetic and environmental factors are implicated in etiology (1). DI may occur due to various factors like rapid proliferation of a part of the internal enamel epithelium, focal failure of growth of the internal enamel epithelium, deep folding of the foramen coecum during tooth development, genetic factors, infection, trauma etc (5).

A total 12 teeth having a combination of TC and DI in 8 patients are reviewed. ( Table 1) In the summarized cases, the age ranged from 7 (2) to 25 (6,8) years with an average of 14.6 years. Male predominance was seen with male to female ratio of 3:1. In the reviewed cases, combination of TC and DI was observed most frequently in the maxillary lateral incisor [n=6] (6-10) followed by the maxillary central incisor [n=4] (1,8) and the mandibular central incisor [n=2] (2,3) with equal predilection for both sides. In the present case, TC and DI were present in the maxillary left lateral incisor of a 23 year old female patient.

All the teeth in the reviewed cases showed type 1 TC [12/12] (1-3,6-10) and majority showed Type II DI [11/12] (1,2,6-10). Similarly in our case, type 1 TC and Type II DI was present. Hattab *et al*. (4) classified talon cusps into 3 types: A] Type 1 [true talon]- is a morphologically well-delineated additional cusp that prominently projects from the palatal surface of a primary or permanent anterior tooth and extends at least half the distance from the CEJ to the incisal edge. B] Type 2 [semi talon]- is an additional cusp of 1 mm or more that extends less than half the distance from the CEJ to the incisal edge and blends with the palatal surface or stands away from the crown. C] Type 3 [trace talon] -is an enlarged or prominent cingulum with variations such as conical, bifid, or tubercle-like.

The most commonly used classification of DI was proposed by Oehlers (11) in 1957. He classified DI based on the x-ray appearance into 3 types: A] Type I- a minor form occurring within the confines of the crown and does not extend beyond the amelocemental junction. B] Type II- it invades the root but remains confined as a blind sac. It may or may not communicate with the dental pulp. C] Type III-a form which penetrates through the root perforating at the apical area showing a ‘second foramen’ in the apical or in the periodontal area. There is no immediate communication with the pulp. The invagination may be completely lined by enamel, but frequently cementum will be found lining the invagination.

Both TC and DI can cause a variety of clinical problems. Presence of TC may result in occlusal interference, accidental cusp fracture, compromised esthetics, irritation to the tongue during speech and mastication. Also, developmental grooves on the TC trap plaque and increase susceptibility to caries and pulpal involvement (2,3,9). DI is typically lined with a thin layer of defective or discontinuous enamel and dentine which allows the penetration of irritants and microorganisms from the saliva directly into the pulp. This often leads to the development of pulpal and periapical pathology (1-3,5,9). In most of the reviewed cases patients were asymptomatic [6/8] while in 2 cases it caused irritation to the tongue, fistula, and periapical pathology (1,2). Our patient presented with swelling and had associated radicular cyst.

Clinical management of TC and DI varies from case to case depending on the symptoms and associated clinical problems (9). Periodic gradual grinding of TC followed by topical fluoride application can be done, if it interferes with occlusion or irritates tongue (2). When there are no signs of pathosis, placement of pit and fissure sealants/restorations in developmental grooves of TC and lingual pit of DI followed by regular observation is recommended (1,3,5). Sealing can be done with GIC (10) or flowable composite (2,7). When pulpal or periapical pathology is present, root canal therapy is required. When pulp necrosis occurs before root-end closure, apexification procedures with calcium hydroxide or mineral trioxide aggregate [MTA] (1) may be necessary. Extraction is indicated only in teeth with severe anatomical irregularities that cannot be treated non-surgically or by apical surgery, and in supernumerary teeth (5).

In the present case, as there was periapical infection, after completing root canal treatment of lateral incisor, enucleation of cystic lesion was done followed by placement of PRF in the surgical defect. PRF was prepared as described by Harish *et al*. (12). Use of PRF resulted in faster healing of the surgical defect.

## Conclusions

A rare case of coexistence of two TC and two DI in maxillary left lateral incisor of a 23 year old female is reported. Such a combination increases the risk of periapical infection in associated tooth. Hence, regular follow up of these cases is important and in cases of associated radicular cysts PRF may be used to speed up the healing of surgical defect.
